# Functional classification of 15 million SNPs detected from diverse chicken populations

**DOI:** 10.1093/dnares/dsv005

**Published:** 2015-04-29

**Authors:** Almas A. Gheyas, Clarissa Boschiero, Lel Eory, Hannah Ralph, Richard Kuo, John A. Woolliams, David W. Burt

**Affiliations:** The Roslin Institute and Royal (Dick) School of Veterinary Studies, University of Edinburgh, Easter Bush Campus, Midlothian EH25 9RG, UK

**Keywords:** SNP, next-generation sequencing, chicken, selection signature, functional variants

## Abstract

Next-generation sequencing has prompted a surge of discovery of millions of genetic variants from vertebrate genomes. Besides applications in genetic association and linkage studies, a fraction of these variants will have functional consequences. This study describes detection and characterization of 15 million SNPs from chicken genome with the goal to predict variants with potential functional implications (pfVars) from both coding and non-coding regions. The study reports: 183K amino acid-altering SNPs of which 48% predicted as evolutionary intolerant, 13K splicing variants, 51K likely to alter RNA secondary structures, 500K within most conserved elements and 3K from non-coding RNAs. Regions of local fixation within commercial broiler and layer lines were investigated as potential selective sweeps using genome-wide SNP data. Relationships with phenotypes, if any, of the pfVars were explored by overlaying the sweep regions with known QTLs. Based on this, the candidate genes and/or causal mutations for a number of important traits are discussed. Although the fixed variants within sweep regions were enriched with non-coding SNPs, some non-synonymous-intolerant mutations reached fixation, suggesting their possible adaptive advantage. The results presented in this study are expected to have important implications for future genomic research to identify candidate causal mutations and in poultry breeding.

## 1. Introduction

With the advent of next-generation sequencing (NGS), we have seen a surge of discovery of millions of genetic variants, particularly single-nucleotide polymorphisms (SNPs), from different species. Characterization of these variants with the goal to delineate those with potential functional implications (pfVars) is often challenging, especially in the absence of comprehensive annotation of genomes for functional elements. Today for some model species, e.g. chicken, the genomes are well annotated for protein-coding regions. As a consequence, our ability to characterize coding variants is much more mature. Additionally, many methods are available to predict the effects of amino acid (AA)-altering variants on protein functions by using evolutionary and biochemical information.^1–4^ These resources have allowed extensive study of the effects of protein-coding variants on disease and other traits.^5^ The protein-coding regions, however, occupy only a small portion of the genome (∼1.5%) while the rest is non-coding.^6^ In spite of this, biological research on non-coding mutations has been limited partly due to the difficulty in interpreting their effects and partly due to the poor annotation of most genomes for functional non-coding elements (fNCEs). The situation, however, is improving rapidly. For instance, a great deal of insight on fNCEs has been generated by the human ENCODE project.^7^ The importance of non-coding variants is further highlighted from the evidence gathered from meta-analysis of genome-wide association studies, which found that 88% of the trait-associated mutations are non-coding.^8^ Complex traits, in particular, are overwhelmingly associated with non-coding mutations, suggesting that these mutations have primarily regulatory roles.^9^ Some predictive models have been developed to assess the effects of SNPs on secondary structures of RNAs, as structural characteristics are often crucial for the functioning of various non-coding RNAs (ncRNA) and *cis*-regulatory elements in mRNAs.^10–13^

Due to scarcity of information on functional elements, particularly the fNCEs, researchers have often adopted evolutionary analyses as a means to locating these elements in genomes, in which, regions conserved across species are assumed to be under purifying selection and functionally important.^14,15^ Mutations within such regions are therefore considered deleterious.^4^ Although these approaches are able to identify regions under purifying selection, they are not suitable for detecting regions evolving rapidly^16^ that are subject to recent positive selections. A number of approaches based on allele frequency spectra and linkage disequilibrium (LD) structure have been proposed for detecting signatures of positive selection by looking for regions exhibiting local fixation, unusually long LD and/or population differentiation that appear in a non-neutral manner compared with the rest of the genome.^17,18^

The aim of the present study was to characterize millions of SNPs detected from the chicken genome in a large NGS project with the goal to identify pfVars. A number of complementary approaches were used to delineate pfVars from both coding and non-coding regions: (i) annotation of the variants against known genes, (ii) predicting effects of the AA-altering variants on protein function, (iii) predicting effects of variants on the secondary structures of mRNAs and ncRNAs, (iv) finding variants that coincided with most conserved elements and (v) detecting regions of local fixation as putative signatures of recent positive selection and considering the fixed pfVars within these as sources of candidate causal mutations of various phenotypes. Since chicken is a major farm animal and an important model organism for genetic and genomic studies, detection and characterization of genetic variants, especially the pfVars, have major incentives for and implications in both research and breeding.

## 2. Materials and methods

### 2.1. Resequencing of chicken samples

Details about the sequenced samples, method of sequencing and alignment of sequence reads to reference genome can be found in a previous study by Kranis *et al.*^19^ In brief, 243 chickens were sequenced and these originated from 24 lines: four commercial broilers (B1–B4), six commercial white egg layers (WEL 1–6), five commercial brown egg layers (BEL 1–5), eight experimental inbred layers (I1–8) and one unselected layer line (RI-J). For 23 lines, DNAs from 10–15 individuals were pooled for creating the libraries, whereas for a single line (WEL6) three individuals were sequenced separately. Sequencing was performed on Illumina GAIIx platform using a paired-end protocol and the sequencing reads were mapped to the reference genome using the Burrows–Wheeler Aligner (BWA)^20^ v0.5.7 using default setting.

### 2.2. Variant calling

Variant calling was performed using the ‘*mpilup’* function of Samtools^21^ (v0.1.18) package. The minimum thresholds for base and map qualities were set to Phred-based scores of 20. Following the initial calling of SNPs, filtrations were performed using the criteria: (i) SNP quality (SNPQ) ≥40, (ii) coverage at SNP position ≥5 and ≤ mean line coverage ± 3SD (Standard deviation), (iii) evidence of alternative (ALT) allele supported by at least two reads: one on forward strand and one on reverse, and (iv) distance between adjacent markers is >1 base. Regions with too high density of SNPs (>10 SNPs/50 bases) were excluded. The coordinates of the SNPs reported in this paper are on the latest chicken reference genome build, *Gallus_gallus_4.* Further details on variant calling are provided in Supplementary Information. All the filtered SNPs have been submitted to the dbSNP with the Handle ID ‘DWBURT’.

### 2.3. Estimation of false discovery rates

False discovery rates (FDRs) of variant calls for the present study were estimated by calling SNPs from Sanger sequencing of 25 random genomic regions and comparing those with SNPs detected from the NGS data from the same regions (further details in Supplementary Information). For each region, only the part that had good-quality Sanger sequence in each individual was used for FDR estimation, and as a result, the lengths of the sequences spanned between 150 and 500 bp.

Based on the comparison of variants from Sanger and NGS data, a SNP was called true positive (TP) when it was detected by both the method, a false positive (FP) when it was detected only by NGS and a false negative (FN) when it was detected only by Sanger. Any sequenced bases that were not called as SNPs by either of the two methods were considered to be true negatives (TNs). Based on these, sensitivity or true positive rate (TPR) was defined as the proportion of actual SNPs that were detected by NGS and was calculated as: TP/(TP + FN). Specificity was defined as the proportion of actual non-SNPs not detected as SNPs by NGS and calculated as: TN/(TN + FP). FP and FN rates were calculated as (1−specificity) and (1−sensitivity), respectively. The proportion of FP SNPs in the NGS data at different filtration criteria was calculated as: FP/(total NGS SNPs retained by the criteria).

### 2.4. Functional annotation and characterization of SNPs

The 15 million SNPs were annotated against the chicken gene database from ENSEMBL (release 71) and most conserved element (MCE) database from UCSC using the software package ANNOVAR (version July 06, 2012).^22^ The MCEs on chicken genome were predicted by PhastCons package by aligning the genomes of six distant species, namely, human, mouse, rat, oppssum, *X. tropicalis* and zebrafish to chicken genome.^23^ To annotate against the MCEs, the co-ordinates of the SNPs were first converted to *Gallus_gallus_3* genome build as the MCEs are mapped onto this build.

The effects of non-synonymous SNPs on protein function were predicted based on evolutionary conservation using the SIFT (jcvi-sift 1.03)^1,2^ and PROVEAN (v1.1.3)^3^ packages. SIFT prediction depends on the degree of conservation at individual AA positions, assuming that functionally important positions have been conserved over evolutionary timescale. Using multiple alignment of homologous but distantly related peptide sequences, SIFT calculates normalized probabilities (SIFT score) of observing all possible AA residues at a position. If the SIFT score of an altered AA is below certain threshold, the variant is considered evolutionary intolerant (INTOL) while above the threshold the variants are considered tolerant (TOL). In contrast, PROVEAN computes an unbiased averaged delta alignment score from multiple alignments of homologous but distantly related peptides as a metric to predict the effects of coding mutations. Below certain threshold of delta score, the variant is declared INTOL. For the present study, SIFT was run using locally generated peptide alignments consisting of sequences from the UniRef90 non-redundant peptide database and predicted sequences from additional 47 bird genomes recently generated by BGI. Sequences were filtered to remove those peptides with over 90% sequence identity to a representative sample using the CD-HIT package and then aligned using Muscle (v.3.8.31). Default scores (<0.05 for SIFT score and >3.25 for conservation score) were used to classify the SNPs as either evolutionary intolerant (INTOL) or tolerant (TOL). PROVEAN was run with the default parameters on the NCBI non-redundant protein database. CD-HIT package was used to remove redundancy and Muscle package was used to generate the alignment. Default threshold (−2.28) of the delta score—defined as the change in alignment score due to introduction of a mutation—was used to differentiate INTOL variants from TOLs.

The effects of SNPs on the secondary structures of mRNAs and ncRNAs were predicted using RNAsnp^12,24^ package. Predictions were performed using two Modes (1 and 2) using default parameters as detailed in the package. The Mode 1 calculates the base-pair probabilities of the wild-type and mutant RNA sequences using a ‘global folding’ algorithm and computes the structural difference between wild and mutant types using Euclidian distance or Pearson's correlation co-efficient for all sequence intervals or local regions. As outputs, the interval with maximum base pairing distance (*d_max*) or minimum correlation co-efficient (*r_min*) is reported along with the corresponding *P*-values. The Mode 2, on the other hand, uses a ‘local folding’ algorithm to calculate base pairing probabilities and is designed to predict the effects of SNPs on large RNA sequences. In a two-step process, this mode first calculates the structural difference using Euclidian distance for all sequence intervals of fixed window length. In the second step, the interval with maximum base pair distance and the corresponding *P*-value is reported. The *P*-values were corrected for multiple testing using Benjamini–Hotchberg (B–H) method.^25^

Ingenuity Pathway Analyses (IPAs) were performed for the genes harbouring potentially deleterious sets of SNPs to gain insight into the biological pathways that are affected. For these analyses, the genes with target group of SNPs were set as up-regulated molecules against the rest of the chicken genes. B–H multiple testing correction was performed and pathways were considered to be enriched with target molecules only when they remained significant (at *P* ≤ 0.05) after the correction.

Allele frequency of each SNP was estimated based on the proportion of good-quality reads supporting the alternative or non-reference allele. Mean frequencies of individual variants were calculated based on the frequency estimates from the populations where the SNP was detected.

### 2.5. Selection signature analysis

Analyses to detect signatures of selection were performed by calculating pooled heterozygosity (Hp) from autosomal SNPs following the method described by Rubin *et al.*^26^ Hp values were calculated for sliding windows of 40 kb size with step size of 20 kb using the following formula.

$$\text{Hp}=\frac{2\sum \text{nMAJ}\sum \text{nMIN}}{{\left(\sum \text{nMAJ}+\sum \text{nMIN}\right)}^{2}}$$

Here ∑nMAJ and ∑nMIN are the sum of major and minor allele counts within a window, respectively. Only windows with >10 SNPs were analysed. BEDTools v2.17.0 was used for creating and intersecting the windows with SNP data, and Hp calculation was performed using custom scripts. The Hp calculation was performed within each commercial broiler (B1–B4), BEL (BEL1–BEL5) and WEL (WEL1–WEL5) lines where pooled samples were sequenced. Significance cut-offs for the Hp values were decided empirically based on chromosome-wise permutation analysis following the methods described by Churchill and Doerge^27^ and Qanbari *et al.*^28^ for individual lines. For the permutation tests, the allele count data were shuffled for 10,000 times within chromosomes, but the SNP positions were kept fixed.

## 3. Results and discussions

### 3.1. Detection of SNPs and FDRs

Initial calling of variants using the criteria of base quality ≥20 and map quality ≥20 detected ∼48 million putative SNPs, which were either segregating or fixed for a non-reference or alternative allele within a line. Upon filtration based on several criteria viz. minimum SNPQ, acceptable coverage, number of reads supporting the alternative allele, inter-marker gap and removal of regions with too high density of variants—over 15 million (*n* = 15,310,407) high-quality SNPs were retained. These formed the basis of subsequent analyses described in this paper. About 48% of the 15 million SNPs are common with known variants in dbSNP (build140) which currently contains ∼8.9 million SNPs for chicken.

The filtered variants were distributed in the genome with an average density of 15 SNPs (± 9.54) per kb or 1 SNP per every 68 bases, although the number of variants in each 1 kb window varied widely ranging from 0 to 112. The number of SNPs detected from each line varied between 3.6 and 5 million. The proportions of segregating variants within lines varied widely ranging from 8 to 77%. For inbred lines, this proportion was particularly low with an average of 21% while the corresponding averages for the commercial lines were ∼74% for broilers, 53% for WELs and 48% for BELs. The smaller proportion of segregating SNPs in the inbred lines was expected as these lines have been developed through many generations of close-mating.

**Figure 1.**
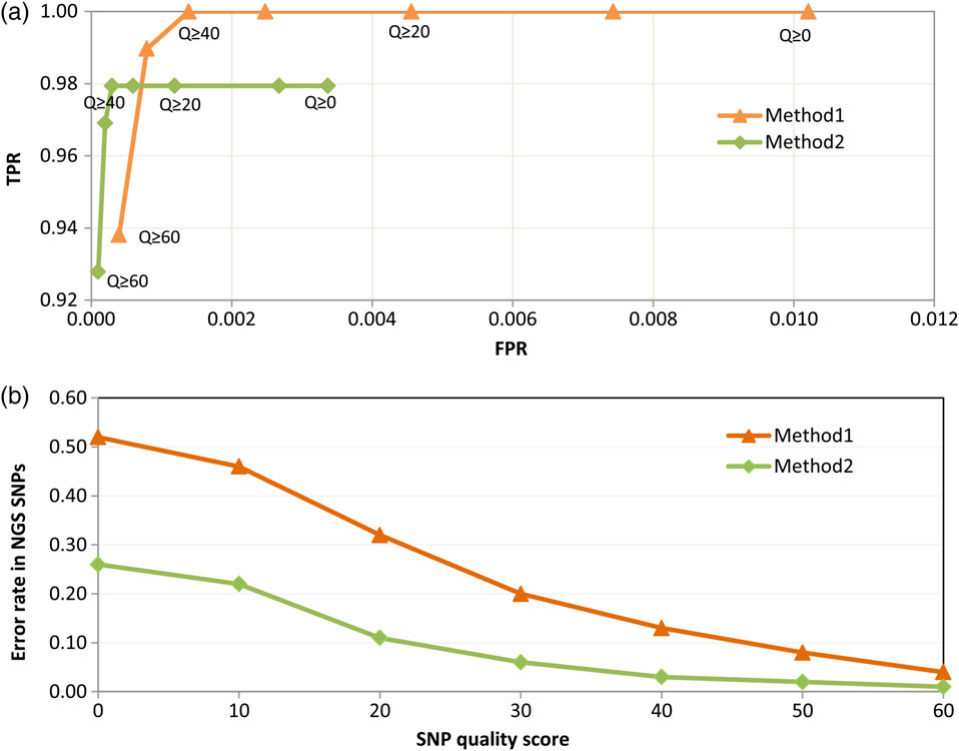
Comparison of two methods of filtration on false discovery rates (FDRs) of SNP call. Method 1 represents filtration based on SNP quality scores (SNPQ) alone and Method 2 represents filtration based on SNPQ and evidence of non-reference or alternative allele by at least one forward- and one reverse-stranded reads. (a) ROC (receiver operating characteristic) curve plotting the true positive rate (TPR) against false positive rate (FPR). TPR is the sensitivity and is defined as the proportion of actual SNPs detected by Next Generation Sequencing (NGS). FPR is (1−specificity) and is defined as the fraction of non-SNP bases wrongly called as SNP by NGS. (b) Error rates or proportion of false positives (FPs) within NGS SNP set at different SNPQ. Both the figures indicate Method 2 as a better filtration approach in terms of FDR.

Even though stringent filtration criteria are often applied to minimize the FPs from NGS data, uncertainties in variant calling may still persist due to alignment errors, inaccurate base calling and insufficient coverage. FDRs for the present study were, therefore, estimated by comparing SNP calls from Sanger Sequence of 25 random regions with that from NGS for the same regions. In total, 97 SNPs were detected from Sanger sequencing of the 11,801 bases while the number of NGS variants from these regions varied depending on the stringency of filtration criteria used (Supplementary Table S1). Before filtration, there were 200 NGS SNPs including all the 97 SNPs detected by Sanger. The Fig. 1a and b compare the FDRs of two filtration methods: (i) using only the SNPQ as filtration criterion and (ii) using SNPQ in combination with the criterion of support of alternative allele by at least two reads—one on the forward strand and one on the reverse strand. Figure 1a shows the trade-off between the FPRs and TPRs for the NGS data using Sanger sequencing as the gold standard. It emphasizes several points. First, the TPRs were very high for NGS although not perfect when high-quality scores were imposed. Second, the FPRs were small in relation to the number of sequenced bases and reduced as higher quality scores were imposed. Third, filtration using Method2 was more effective than Method1 in reducing the FPR.

Figure 1b compares the error rates for the NGS SNPs between the two filtration methods mentioned above. It shows that when the criterion of support of alternative allele by at least one forward- and one reverse-stranded reads alone was applied without any filtration on SNPQ (i.e. when SNPQ = 0), the error rate dropped drastically from 51 to 26%. At SNPQ ≥ 40, imposing this criterion reduced the error rate from 13 to 3% but at the same time increased the FNR (Supplementary Table S1). This error rate of 3% in detecting SNPs from NGS data is comparable to the rates reported in other studies such as 4.5% reported for chicken^26^ and 2.6% for turkey.^29^

Prediction of FNR, however, is more difficult as it may be affected by a range of factors such as missing regions in the genome assembly where variant calling was not possible, extent of repeat regions in the genome, presence of regions that are difficult to sequence due to their biochemical properties and attempt to reduce FPs resulting from alignment artefact due to presence of indels or duplicated regions. As a consequence, even though the above analysis estimated FNR to be only 2% with Method 2 (Supplementary Table S1), the actual rate is expected to be much higher. Since the goal of the present study was to characterize a set of high-quality SNPs by minimizing the FPs, the application of stringent criteria was justified even with some compromise on the FNR.

### 3.2. Coding and non-coding variants

Only 2.2% of the 15 million SNPs was predicted to be within protein-coding regions and 1.2% as AA-altering (non-synonymous and stop-gain/loss) when annotated against the ENSEMBL gene database for chicken. The rest of the SNPs were classified as non-coding (Table 1). Apart from the AA-altering variants, the other potentially functional categories included: variants with the potential to disrupt splicing events (0.09%); variants in 3′and 5′ UTRs with the potential to regulate protein translation (1.3%); those within 1 kb up- or downstream of transcription start or end sites with possible roles on transcriptional regulation (2.8%); and finally, the SNPs belonging to ncRNAs (0.02%). In addition to the ENSEMBL gene database, all the SNPs were checked against 1,608 ncRNA transcripts, which have recently been characterized.^30^ A total of 2,062 (0.01%) variants fell within these transcripts: 131 in CD-snoRNAs, 155 in HACA-snoRNAs, 1,073 in microRNAs, 241 in regulatory regions, 82 in tRNAs, and the remaining 380 were in unclassified transcripts. Some of these ncRNA variants (*n* = 284) were previously annotated to be within protein-coding genes (exonic, UTR and intronic categories) when the ENSEMBL database was used. Major proportions of these SNPs with dual annotation were enriched for three ncRNA categories: regulatory region (34% of the 284), microRNA (27%) and tRNA (14%) types. Such overlaps could be due to potential difficulty in distinguishing ncRNA from mRNA or might truly reflect regions having dual functions as both non-coding and coding.^31^

**Table 1.** SNPs belonging to different annotation categories

Annotation categories	Number (%)^a^	Mean AAF (± S.D.)^b^	No. detected from >10 lines with mean AAF > 0.9^c^	No. detected from all 24 lines with mean AAF > 0.9^c^	No. of private SNPs with AAF > 0.9^c^
Intergenic	7,688,980 (50.22)	0.63 (0.24)	584,504 (7.60%)	223,114 (2.90%)	172,139 (2.24%)
Up/downstream	433,726 (2.83)	0.60 (0.25)	31,710 (7.30%)	10,348 (2.38%)	8,377 (1.93%)
Intronic	6,637,278 (43.35)	0.63 (0.24)	503,093 (7.57%)	190,587 (2.87%)	134,161 (2.02%)
Exonic	335,331 (2.19)	0.46 (0.26)	15,686 (4.48%)	5,846 (1.67%)	3,293 (0.94%)
Non-synonymous INTOL	88,655 (0.58)	0.28 (0.16)	416 (0.47%)	129 (0.15%)	288 (0.32%)
Non-synonymous TOL (or no prediction)	93,596 (0.61)	0.42 (0.25)	4,114 (4.38%)	159 (1.70%)	850 (0.90%)
Stop-gain/loss	1,071 (0.01)	0.38 (0.24)	21 (1.96%)	7 (0.65%)	11 (1.03%)
Synonymous	151,999 (0.99)	0.58 (0.25)	11,135 (7.31%)	4,116 (2.70%)	2,144 (1.41%)
Splicing	13,147 (0.09)	0.35 (0.22)	319 (2.42%)	115 (0.87%)	73 (0.55%)
UTR3′/UTR5′	204,693 (1.34)	0.58 (0.25)	14,299 (6.98%)	4,974 (2.43%)	3,504 (1.71%)
ncRNA	3,122 (0.02)	0.54 (0.28)	136 (4.36%)	47 (1.51%)	88 (2.82%)
Within MCE	537,966 (3.51)	0.55 (0.26)	35,856 (6.66%)	13,997 (2.60%)	7,721 (1.43%)

^a^The percentages are in relation to 15 million SNPs.

^b^AAF refers to Alternative allele frequency.

^c^The percentages are in relation to the number of SNPs within the annotation category.

The AA-altering SNPs (*n* = 183,320) were detected from 13,286 genes representing 74% of the known genes in chicken. Their density in terms of length of coding sequence varied widely across the genes ranging from 1 SNP per 19 bases to 1 per 731 kb (mean 1 SNP per 5.5 kb). A high degree of correlation (*r* = 0.75, *P*<0.001), however, was observed between the densities of AA-altering and synonymous mutations. This correlation combined with the large variation in the densities of coding SNPs indicate that some genes are more prone to accumulate variants either due to differential mutation rates or uneven selection pressure.^32^ IPA demonstrated that the genes with high density of AA-altering variants (>10 SNPs/kb coding sequence) were significantly enriched in nine biological pathways (*P* ≤ 0.05 after correction for multiple testing). These pathways were associated with a DNA replication, recombination and repair, cell-to-cell signalling and interaction, metabolism of lipids, carbohydrates and amino acids, and cancer (Supplementary Table S2). It is difficult to explain why certain genes within important pathways harbour high density of AA-altering variants. One explanation is that not all of these variants were predicted to have radical effect on the function of the genes, and when harmful, they were generally present at low frequencies; these aspects have been further explored in the next two sections. Closer inspection of these genes further reveals that majority of these (∼70%) have one or more paralogs in the genome, indicating that the loss of function of the genes due to deleterious variants may partly be rescued by their paralogous genes. There were, however, 20 genes that showed extreme SNP density (≥50 SNPs/kb coding sequence). While these genes may represent regions with rapid mutation rates, it is possible that the high variant density in these genes is actually a result of error in the reference genome assembly and/or error in mapping of sequence reads due to high level of duplications. Eleven of these 20 genes had no paralog in the genome and may represent assembly errors. The other 9 genes had one or more paralogs, as many as 58, and the high SNP density may be a result of mapping errors.

### 3.3. Negative impact of non-synonymous substitutions on protein function

Even though non-synonymous SNPs change amino acid sequence within a protein, the effects are not always harmful or radical on protein function. Using SIFT, 23% of the non-synonymous variants (*n* = 41,980) were predicted as ‘intolerant’ (INTOL) having radical effect and 51% were predicted ‘tolerant’ (TOL) (Supplementary Fig. S1a). The remaining 26% did not have any predictions either due to lack of sufficient aligned sequences or poor confidence in predictions. Contrary to this, PROVEAN provided predictions for >98% of the SNPs and much higher proportion of the variants (45%; *n* = 82,564) were predicted as INTOL (Supplementary Fig. S1b). This can be attributed to two factors. First, PROVEAN takes into account the sequence context of a variant rather than only individual amino acid position.^3^ Second, in the absence of homologous peptides, PROVEAN can still perform analysis by comparing the alignment of the query sequence to itself before and after the introduction of the mutation, which is not possible with SIFT. Together 49% of the non-synonymous SNPs (*n* = 88,655) were predicted INTOL by at least one of the algorithms while only 20% (*n* = 35,889) were predicted as INTOL by both.

IPA analysis of genes harbouring INTOL non-synonymous SNPs from broiler, WEL and BEL groups suggested many pathways (*n* = 162–211 after multiple testing correction), especially signalling pathways (87–92%), to be significantly enriched for these variants (See the Supplementary Table S3 for the top five pathways from each group). Most of the INTOL variants were found to have low frequency and were detected from few lines only (Supplementary Fig. S2), thereby limiting their harmful effect (see next section for further information on allele frequency). This can explain how important pathways could harbour INTOL variants. IPA analysis on inbred lines found only two pathways as significant of which the ‘Role of *BRCA1* in DNA Damage Response’ was found highly significant (*P* < 0.001; with 20 of the 71 genes passing the cut-off criteria). This pathway consists of genes encoding for tumour suppressors and DNA damage repair proteins. Since, the inbred lines have been bred for experimental purposes for susceptibility/resistance to different viral pathogens, such as Marek's Disease Virus (MDV), Avian Leucosis Virus (ALV) and Lymphoid Leucosis Virus (LLV), it is likely that these genes within these pathways are candidates for explaining the susceptibility/resistance towards these pathogens.

### 3.4. Effects of SNPs on RNA secondary structure

The functions of many ncRNAs and *cis*-regulatory elements in mRNAs often rely on their distinctive secondary structures.^12^ Previous studies have shown that mutations within untranslated regions (UTR) of mRNA transcripts can alter the structures of *cis*-acting regulatory elements that reside within these regions and thereby affect the translational and transcriptional efficiency and stability of mRNAs.^33,34^ Even apparently silent synonymous mutations have been shown to affect mRNA folding and stability, altering protein translation.^35^ Therefore, in the present study, the putative effects of all UTR, synonymous, non-synonymous and ncRNA variants on the secondary structures of RNAs were investigated using the RNAsnp package.

About 9% (*n* = 19,296) of the UTR, 11% (*n* = 19,603) of the non-synonymous, 8% (*n* = 12,001) of synonymous and 7% (*n* = 218) of the ncRNA variants were predicted to affect RNA secondary structure by at least one of the parameters and models used (*P* ≤ 0.05 before correcting for multiple testing) (Supplementary Table S4). Although after correcting for multiple testing, only 15 variants (4 UTR3′, 7 ncRNA and 4 non-synonymous) remained significant (*P* ≤ 0.05), the other variants, however, can still be considered suggestive and be used for shortlisting candidate functional variants. These variants including the suggestive ones were located within 12,296 protein-coding genes and 176 ncRNA transcripts.

### 3.5. SNPs within evolutionary conserved elements

Genomic regions conserved across distantly related species are assumed to be under purifying selection, and hence variants within these regions are likely to be harmful. The 15 million SNPs were annotated against the 950,084 known ‘most conserved elements’ (MCEs; UCSC^23^), covering 6.5% of the genome and with their sizes varying from 1 base to 4.3 kb (median 43 bases).

About 3.5% (*n* = 537,966) of the 15 million variants fell within only 2.5% of the MCEs, while the rest of the conserved elements were devoid of any SNPs. The density of variants within MCEs was 8 SNPs/kb, which was much lower compared with the genome-wide density of 15 SNPs/kb, supporting their conserved nature. Major proportions of the MCE variants belonged to intronic (38%), exonic (33%) and intergenic (23%) categories while the remaining 5% fell within other categories such as UTR (2.2%), up/downstream (1.8%) and splicing (0.9%) and ncRNA (0.1%). Among the MCE-exonic variants, 51% (*n* = 91,789) were AA altering and majority of these (*n* = 60,016) were predicted as INTOL as was expected due to the evolutionary conserved nature of these regions.

### 3.6. Allele frequency distributions of SNPs in different annotation categories

**Figures 2.**
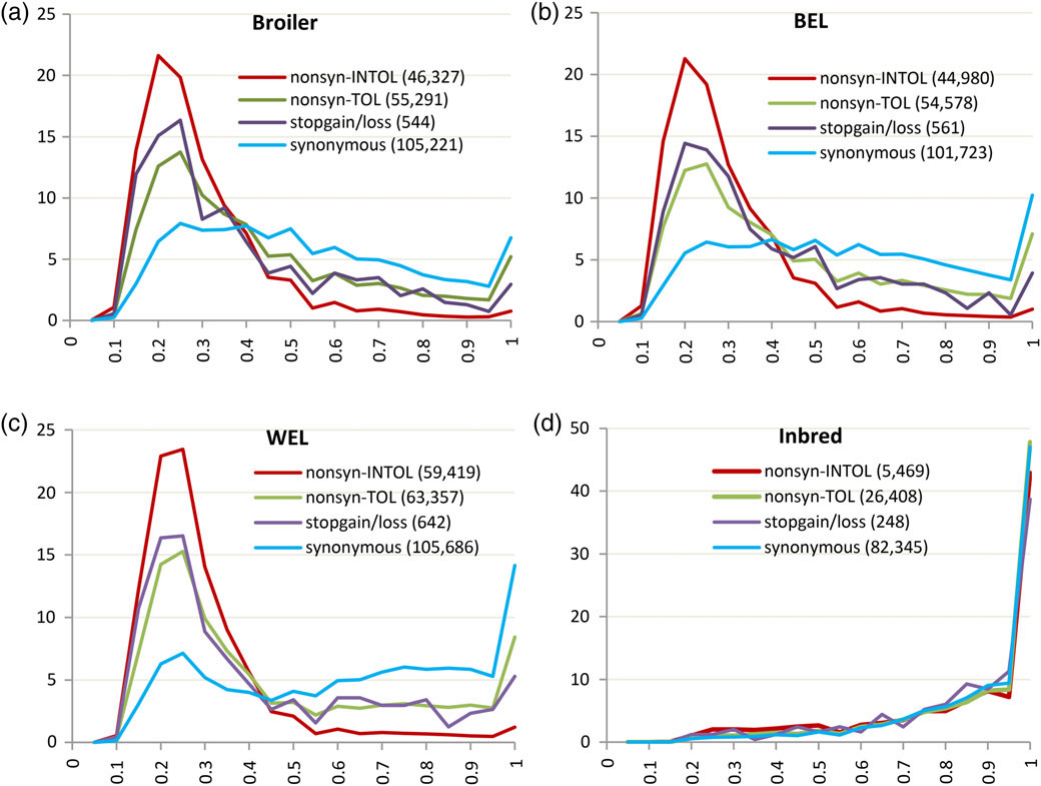
(a–d) Frequency distributions of alternative alleles of SNPs from protein-coding regions. *X*-axis represents the allele frequency (ranges from 0 to 1) and *Y*-axis represents percentage of SNPs within a category. For the broiler, brown egg layer (BEL) and white egg layer (WEL) groups, the *Y*-axis is scaled from 0 to 25 (in percentage), and for Inbred group, the *Y*-axis is scaled from 0 to 50 (in percentage).

**Figures 3.**
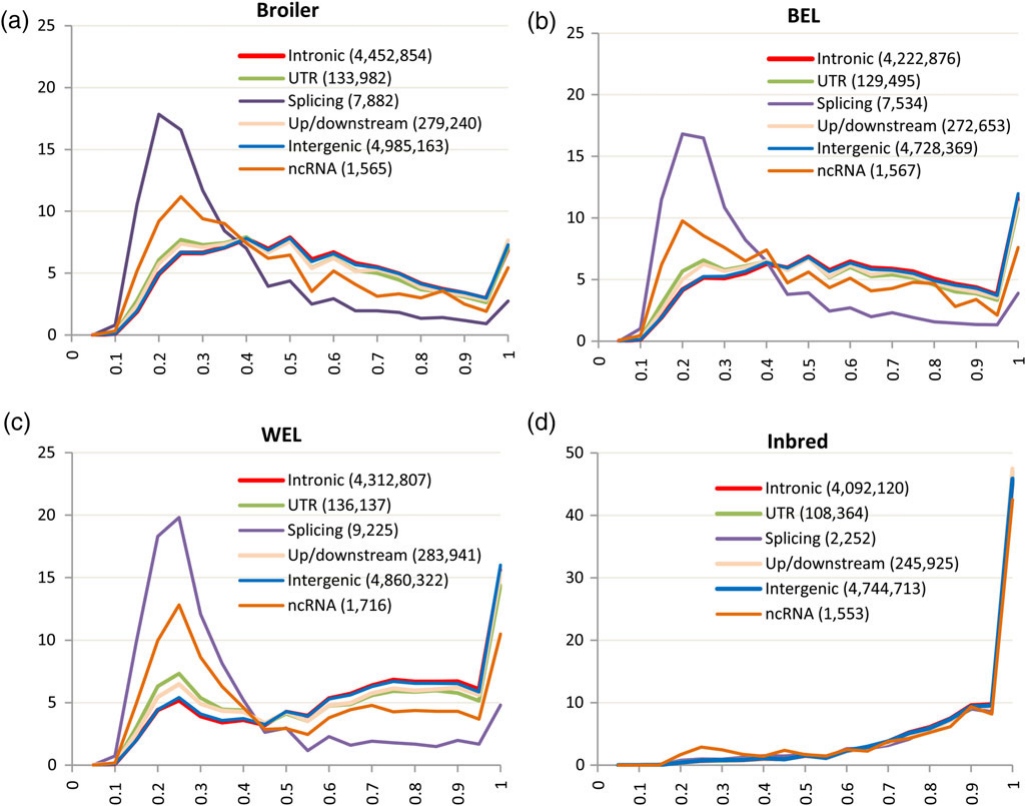
(a–-d) Frequency distributions of alternative alleles of SNPs from intergenic, intronic, up/downstream, splicing, ncRNA and UTR categories. *X*-axis represents the allele frequency (ranges from 0 to 1) and *Y*-axis represents percentage of SNPs within a category. For the broiler, brown egg layer (BEL) and white egg layer (WEL) groups, the *Y*-axis is scaled from 0 to 25 (in percentage), and for Inbred group, the *Y*-axis is scaled from 0 to 50 (in percentage).

Frequency distributions of non-reference or alternative alleles (AAF) of pfVar categories were compared with those of potential neutral variants to investigate differences in their patterns (Figs 2 and 3, Table 1). This revealed a number of aspects. First, except in the Inbred group, in commercial chickens the non-synonymous, stop-gain/loss and splicing variants were heavily skewed to the left, i.e. fell within lower frequency bins compared with other SNP categories. About 50–61% of the AA-altering and splicing variants had AAF ≤ 0.3, whereas synonymous, intronic and intergenic variants, which are largely assumed to be neutral, showed more or less even distributions across the frequency bins (Figs 2 and 3). The overall mean frequency for AA-altering and splicing variants was 0.35 (SD: 0.22) compared with 0.58 (SD: 0.25) for synonymous or 0.63 (SD: 0.24) for intergenic and intronic variants (*t*-test, *P* < 0.001). Apart from the AA-altering and splicing variants, ncRNA SNPs also showed a left skew in all commercial groups with ∼33–37% belonging to frequency bins ≤0.3.

Second, although both non-synonymous INTOL and TOL variants showed left-skewed distribution, much greater proportion of INTOL SNPs (69–73%) fell within lower frequency bins (≤0.3) compared with TOLs (43–47%) in the commercial groups. This pattern provides confirmation to the assumption that INTOLs are more deleterious than the TOLs, but this does not equate the TOL variants with the neutral ones. Rather the left-skewed distributions of both TOL and INTOL variants indicate that non-synonymous SNPs in general are selected against.

Third, comparison of frequency distribution of SNPs within MCE and non-MCE revealed that only those MCE-SNPs belonging to AA-altering, splicing or ncRNA categories had a marked difference in the allele frequency distribution compared with non-MCE categories with the former group clustering around lower frequency bins (AAF ≤ 0.30) (Supplementary Fig. S3a–d). MCE-SNPs belonging to other annotation categories such as intergenic, intronic, etc. all showed the same pattern of frequency distributions as of neutral categories.

Fourth, within the inbred group, all types of variants, irrespective of their categories, showed very high level of fixation. However, the number of AA-altering and splicing variants in inbred lines were much fewer compared with those from commercial groups (Figs 2 and 3). Especially, the number of non-syonymous INTOL variants in inbred lines was 8–11 times less than those in commercial groups. This observation indicates that the negative selection has prevented highly deleterious mutations from reaching fixation in the inbred lines and the existing INTOL variants are possibly not severely harmful under the environmental conditions these birds are raised.

Finally, Figs 2 and 3 reveal that there were no SNPs within low-frequency bin (≤0.05). This result was expected due to a number of reasons. First of all, the allele frequency in the present study was calculated only based on 10–15 individuals per line. This meant that estimated frequency within line could not be below 0.03–0.05. Second, the sequencing of pooled DNA (Pool-seq) is known to be extremely prone to losing rare and low-frequency variants when performed at a low depth of sequencing coverage.^36,37^ In the present study, the sequence coverage was 8–17X, which was often lower than the number of individuals per pool (10–15) and the corresponding number of chromosomes (20–30) that needed to be sampled. This was compounded by the use of various filtration parameters such as quality scores, minimum coverage and the evidence of non-reference allele in both forward and reverse strands, which resulted in the loss of many true SNPs, especially from low-frequency categories. Cutler and Jensen^37^ predicted that filtering SNPs from Pool-seq data with a Phred quality score of 20 (predicted error rate 1%) would lead to the loss of 29% of the variants when the coverage is between 11 and 50X, and majority of these variants will be rare and of low frequency. They also showed that if the error rate was very low (e.g. 0.1%) and only a small number of samples were pooled (e.g. 10 samples) that would require sequencing to a depth of 300X to detect all the variants including the rare ones.

The proportions of pfVars that appeared in multiple lines (>10 lines) at high frequencies (>0.9) were investigated as these might represent variants with adaptive advantage (Table 1). Alternatively, this is also possible that these variants are only moderately deleterious or neutral and have increased in frequency through genetic drift or by hitchhiking with linked variants under positive selection. A very small proportions (0.5–2%) of stop-gain/loss, INTOL and splicing variants were detected from multiple lines at high frequency, while for categories like synonymous, intergenic, intronic, up/downstream and UTR, the corresponding proportions were much higher, above 7% (Table 1). This agrees with the general expectation that the former group of variants are selected against due to their potential harmful effects. Since stop-gain variants result in the premature termination of proteins, they were further investigated which revealed that in most cases (85%) they were located: (i) in genes that have other paralogs in the genome, which might minimize their impact (64%); (ii) in uncharacterized hypothetical genes for which no functions are yet known and these genes may be prediction artefacts (25%); (iii) near the end of coding sequence thus allowing the translation of at least 90% of the length of the protein retaining most of its functionality (16%) or at the very beginning of the protein (12%) (Supplementary Fig. S4). A similar observation was reported by Ng *et al.*^38^ Yet there were a few stop-gain variants that did not conform to any of these rules, yet were detected at very high frequencies (>0.9) from multiple lines (>10). These included three stop-gain mutations from the genes: *C1ORF101* (Chr3_34083273), *CCDC60* (Chr15_9679384) and *GLOD4* (Chr19_6926808). Their relative position within the respective proteins (calculated by dividing the position of the variant with the protein length) varied from 0.33 to 0.67. These variants may therefore have some selective advantage in domesticated chicken lines. Similarly, over 80% of the INTOL variants detected from multiple lines at high frequencies were located within genes with one or more paralogs or in novel genes with unknown function as discussed above.

Over 14% (*n* = 2,248,437) of the 15 million SNPs were private to individual lines. Of these, only 12.5% SNPs were fixed or nearly fixed. Although much smaller proportions of private fixed variants belonged to AA-altering and splicing categories (Table 1), these could be important for the lines in question.

### 3.7. SNPs within regions of selection signature

One of the signatures of positive selection is that it creates regions of local fixation compared with the overall pattern of diversity in the entire genome—a phenomenon known as a ‘selective sweep’.^17^ The regions of local fixation were investigated by calculating pooled heterozygosities (Hp) in sliding windows using the SNP data for individual broiler, BEL and WEL lines. Since only the windows with >10 SNPs were analysed, the number of analysable windows varied from 44,237 to 45,378 per line (Supplementary Table S5). The average Hp values varied not only among the lines (0.22–0.36) but also among chromosomes (0.16–0.43), which is likely due to the wide variation in recombination rates among chromosomes.^39^ Therefore, in deciding the threshold Hps for detecting putative selective sweep (pSS) regions, chromosome-specific permutation analysis was performed within each line. Proportion of the windows with fixation signal varied considerably across the lines ranging from 0.7 to 9.8% at *P* < 0.001 while with relaxed thresholds (at *P* < *0.01* or *P* < *0.05)*, the number increased considerably.

Differentiating the signatures generated by true selective sweep is challenging, because similar effects might be left on the genome by demographic events and population structure.^17^ While the permutation test improves the confidence in detecting pSS, comparison of the signals across populations can further confirm the results and minimize the incidence of false signals from the confounding effects of demographic events.^18^ Therefore, in the following sections, we focus only on the common windows that passed critical Hps (at *P* < 0.05) in all the lines within a group (broiler, BEL or WEL) or across groups. Sex chromosomes were excluded from analyses to avoid interpretational problems as the lines were differentially consisted of either male or female samples.

Common signals were detected only from 143 widows in broilers, 163 in BEL and 49 in WEL. Many of these windows were adjacent or overlapping and hence were joined to obtain non-overlapping regions that resulted in 60 discrete pSS regions for broiler, 66 for BEL and 25 for WEL (Supplementary Fig. S5). The relatively lower number of shared regions in the WEL group was due to a single line—WEL5, which elicited very few fixation signals (only 4% windows compared with ∼14% in other WEL lines). This is due to the fact the WEL5 had higher level of fixation relative to other lines and as a result had low critical Hps from the chromosome-wise permutation analyses causing very few windows to elicit selective sweep signal. If WEL5 is excluded from the comparison, 208 windows and 100 non-overlapping pSS regions are found to be shared among the other four WEL lines. Across different groups, we discovered only seven windows shared between broilers and BELs, five between BEL and WEL (excluding WEL5) and only one shared between broiler and WEL (Supplementary Fig. S5).

As a proof that our approach could detect sweep regions, our results were compared with previous studies. This demonstrated that many of the genes that were partly or fully covered within our pSS regions had also been detected by other studies. Some notable genes among these are the following: *BCDO2* (β-carotene oxygenase 2) that has been found associated with yellow skin in domestic chickens;^40^ *TSHR* (thyroid-stimulating hormone receptor) playing important roles in regulation of metabolic functions and reproduction in commercial chickens;^26^ *IGF1*(insulin-like growth factor-1) and *HNF4G* (hepatocyte nuclear factor 4 gamma) which are candidates for growth;^26,41^ *PMCH* (pro-melanin concentrating hormone) that plays important role in regulating appetite and metabolic functions;^26,42^ *INSR* (insulin receptor), which affects growth traits with a central role in insulin signalling;^26^ and *NELL1*(*NEL-like 1*) gene, potentially associated with skeletal integrity in broiler.^41^ Moreover, over 91% of the pSS regions also overlapped with one or more QTLs (in chicken QTLdb) associated with production, behavioural- and health-related traits such as body weight, growth performance, meat quantity and qualities, fat content, egg production and qualities, feather pecking and antibody response to various pathogens. This provided further support in favour of predicted selection pressure on these regions.

The pSS regions harboured a large number of variants (6K–12K depending on groups), either fixed or segregating. While it is expected that the variants directly under selection will be fixed or be at high frequency (AAF > 0.9), the other functionally active segregating variants within the pSS regions may be selectively advantageous or deleterious and will exert their effects quantitatively on the associated phenotype. About 38–56% of the variants in pSS regions were fixed in different groups. Many of the fixed variants, however, are likely to be neutral which might have reached fixation through hitchhiking just by being closely linked to causal mutations. Therefore, we decided to focus mainly on the pfVars viz. AA-altering, splicing, RNA structure-altering, MCE variants or those in ncRNAs. This resulted in 4,679 variants from all the pSS regions of which only 17% (*n* = 815) were fixed in at least one chicken group and 2% (*n* = 106) were fixed across the groups (Supplementary Fig. S6). We hypothesized that if a window elicited selection signal from more than one group and if variants therein were present ubiquitously and were fixed across the groups, this most likely represented old selection—possibly associated with traits that promote high production rates or better performance as a result of commercialization of the chicken or are relevant to adaptation in domesticated situations. On the other hand, if a pSS region was detected within a single group and variants were fixed only within that group, it was probably associated with group-specific traits. The major findings are discussed in the following sections.

### 3.7.1. Ubiquitously present pfVars in the BCDO2 gene are candidates for yellow skin trait

Yellow skin colour is a ubiquitous feature in all Western commercial chicken.^40^ It has been postulated that in chicken the trait is expressed due to the presence of one or more *cis*-acting and tissue-specific regulatory variants that inhibit(s) the expression of the *BCDO2* gene, which otherwise is responsible for degradation of carotenoid. In a previous study, Eriksson *et al.*^40^ suggested a minimum haplotype of 23.8 kb on chromosome 24 to be shared across diverse domesticated chicken breeds with yellow skin, and this haplotype is expected to harbour the causal mutation for the trait.

In the present study, the 40-kb window (Chr24:6120000–6160000) that covered the minimum haplotype of yellow skin trait elicited fixation signals in all the broiler and BEL lines. Within the WEL group, however, only two of the five lines produced signal for this window and as a result it was not detected as a common pSS region from the WEL group. Closer inspection of the variants within the window revealed that there were several pfVars (*n* = 13), of which seven were located directly within the yellow skin haplotype and were completely fixed across all the commercial lines including the WELs (Supplementary Fig. S6). All of these variants are located either within the *BCD02* gene or nearby this gene and hence can be considered as candidate causal mutations. These include: 1 intergenic-MCE, 3 intronic-MCE, 2 non-synonymous TOL and 1 UTR5′ SNP that was suggestively RNA structure altering. In spite of complete fixation of these variants, the failure to detect a signal for the region from certain WEL lines suggests that the sweep region is smaller than the 40-kb window size used in this study, which is consistent with the size of shared haplotype (23.8 kb). Erriksson *et al.*^40^ speculated that the causal mutation(s) of the yellow skin trait will be within evolutionary conserved region(s) and will be regulatory in nature. Considering these, the variants within MCEs appear to be the most likely candidates.

### 3.7.2. Ubiquitously present non-synonymous SNPs in the TSHR gene are potential causal mutations of a known sweep locus

Two recent studies have, respectively, detected and validated a strong signature of selection overlapping the *TSHR* gene.^26,43^ Although the first study speculated the gene to be associated with domestication in chicken, the later study found a more recent origin of this signature and concluded that the *TSHR* mutation was neither a pre-requisite nor a critical factor in domestication process; instead it is most probably related to the improvement of chickens that began during the industrial revolution. Although the causal mutation(s) within this region and its phenotypic effects are yet to be established, the biological significance of the *TSHR* gene on the regulation of metabolic activities and reproduction, particularly in extending the mating season, is well known.^44,45^ Rubin *et al.*^26^ detected a non-synonymous variation within the *TSHR* gene that they suspected was the causal mutation.

The present study also detected a strong signal covering part of the *TSHR* gene from the analyses of broilers and BEL lines. Within the WEL group, four of the five lines were significant for the same *TSHR* window but due to failure in getting signal from a single line, it was not ultimately picked up as a common signal. About 62% (*n* = 221) of the variants within the gene had average frequency >0.90 suggesting a very strong selection pressure. There were only two fixed pfVars in this region though (Supplementary Fig. S6): a non-synonymous TOL mutation and a non-synonymous INTOL SNP, the same variant that had also been detected by Rubin *et al.*^26^ This INTOL variant also coincided with an MCE. Both the variants were completely fixed in all the commercial lines except in a single WEL line, although in this line the variants prevailed at high frequency (0.6–0.8). These variants were also fixed in non-commercial lines such as RI-J and inbred lines. These could therefore be the candidate causal mutations.

### 3.7.3. The putative sweep regions show enrichment of non-coding mutations within higher frequency classes

**Figure 4.**
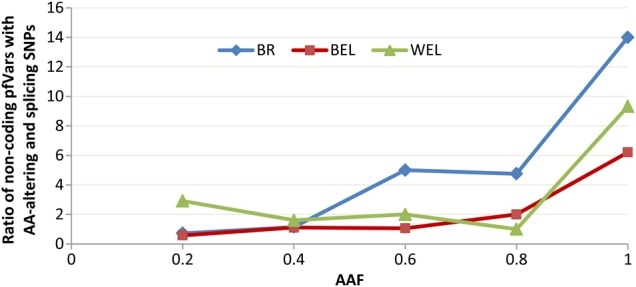
Ratio of non-coding putatively functional variants (pfVars) with AA altering and splicing types at different allele frequency bins within selective sweep regions. The non-coding pfVars included intergenic, intronic, UTR and down/upstream variants from most conserved elements, variants located within known ncRNAs and variants that were predicted to alter RNA secondary structure. The graph indicates that high-frequency variants are enriched with non-coding types. BR, BEL and WEL refer to broiler, brown egg layer and white egg layer groups, respectively.

Plotting the ratio of the number of non-coding and coding variants against allele frequency reveals that there is a significant enrichment of non-coding mutations at higher frequencies compared with coding variants (*P* < 0.001 in *χ*^2^ test) (Fig. 4). This is expected as non-synonymous, splicing or stop-gain/loss variants can have radical impacts on the translated protein, whereas the functional non-coding variants may be much more tolerated due their regulatory roles on gene expression.^9^ This is consistent with the observations from various genome-wide association studies that revealed that majority of the genetic variants associated with complex traits lie within non-coding regions of the genome.^8,9^

Between 37 and 45% of the pSS regions across all the groups were completely devoid of genes and coincided entirely with intergenic regions and 54–70% of all the pSS regions showed fixation of only non-coding variants including intergenic, intronic, up/downstream and UTR SNPs. For instance, some of the genes found within the pSS regions were particularly enriched for fixed intronic pfVars (Supplementary Fig. S6) and most of these genes have previously been detected in other selection signature studies^26,41^ as well, thereby strengthening their likelihood to be within true sweep regions. It is possible that these intronic variants exert their effects through regulation or disruption of transcriptional activities, splicing efficiency of their host genes or expression of alternative transcripts.^46^

Overlaying the known QTLs with the pSS regions reveals that the regions harbouring only non-coding fixed pfVars overlapped with QTLs of complex traits. For example, a large number of pSS regions detected in our study overlapped with QTLs for bone traits such as bone strength, bone-mineral content, bone-density, etc., suggesting a multi-gene hypothesis for regulation of these traits. Most of these pSS regions also coincided with QTLs for other traits as well, which further indicates possible pleiotropic effects of the same genes on multiple traits.^47,48^ Only one of the pSS regions (B-R47 in Supplementary Fig. S5) from broiler lines, however, overlapped with only bone-related QTL(s) viz. Tibia width^47^ and mineral density in femur bone,^48^ thereby allowing its closer inspection. Both the QTLs, however, were detected with only suggestive significance and as a result their effect size is expected to be small, which is an indication of involvement of regulatory causal variant(s). This pSS region overlaps with the *PAPPA2 (Pappalysin 2)* gene. The protein encoded by this gene cleaves the *IGFBP-5* (insulin-like growth factor binding protein 5), which is an important stimulator of bone formation.^49^ Among all the variants detected from and around this gene, only non-coding SNPs reached fixation including 46 intronic, 2 upstream and 37 intergenic variants. Only one of the fixed intronic variant (Chr8:6817157) coincided with an MCE. This particular variant was found to be fixed in all the broiler and WEL lines and also in two of the five BEL lines. It is possible that this is a functional variant with a regulatory role.

### 3.7.4. Evolutionary intolerant non-synonymous SNPs prevailing at high frequencies may confer adaptive advantage

From the pSS regions, 11 non-synonymous INTOL variants were detected that reached fixation in one or more groups. Apart from the one INTOL SNP from the *TSHR* gene that has been discussed above, the other 10 variants were detected from the following genes: *GCNT3* (*n* = 2), *PLA2R1* (*n* = 1), *FOXI1* (*n* = 1), *ZNF507* (*n* = 1), *ADAMTS7* (*n* = 1) and *ENSGALG00000023731* (novel chicken gene; *n* = 4). Even though the pSS regions overlapping these genes were detected from specific groups (either BEL or WEL), a number of these INTOL (*n* = 5) variants prevailed ubiquitously at high frequency or at complete fixation in all or most of the lines irrespective of groups. Some of the variants also coincided with MCEs. Their high frequency implies that even though evolutionary intolerant, these variants possibly confer important adaptive advantages. Classic examples of genetic defects being selected as adaptive advantage includes the prevalence of certain blood-related disorders in malaria-infected regions as a means to confer resistance to the latter disease^50^ and the increase in frequency of the risk allele of diabetes mellitus during food scarcity in the past.^51^ It is, however, also possible that the fixed INTOL variants detected in the present study are not actually beneficial but have been hitchhiked to fixation with linked causal mutation.^52^

Exploring the functions of the above genes harbouring the INTOL variants reveals that they perform a variety of functions which may be related to commercially important traits in chicken. The *GCNT3* (*glucosaminyl N-acetyl transferase 3, Mucin Type*) gene, for instance, plays an important role in the immune system through the synthesis of mucins in mucus-secretary epithelial tissues.^53^ Mucin hyper-secretion can be a common response to many pathogenic infections and cancers.^54^ The proteins encoded by the *PLA2R1* (*phospholipase A2 receptor 1*) gene exert various effects in different tissues including inducement of cell proliferation and production of lipid mediators by serving as a receptor of phospholipase.^55^ The region harbouring this gene overlapped with QTLs associated with several traits most notably for fat deposition, body weights and egg production performance. The *FOXI1* (*Forkhead box I1*) gene codes for a transcription factor that in human is involved in the physiology of several organs viz. inner ear, testis and kidney.^56^ A recent study in human has detected a sweep region covering this gene and predicted that positive selection of the *FOXI1* gene imparts adaptive advantage in kidney-mediated water-electrolyte homeostasis in African population in response to climatic condition.^57^ The pSS region covering this gene, in our study, overlapped with QTL(s) for growth, age-at-first egg and fear-related behaviour. The functions of the *ZNF507* and *ENSGALG00000023731* genes are not yet well studied, although *ZNF507* possibly plays role in transcriptional regulations affecting multiple functions.^58^ The possible effect of the *ADAMTS7* gene and the fixed pfVars therein is discussed in the following section.

### 3.7.5 Broiler or layer-specific variants

About 12% (*n* = 94) of the pfVars detected from various pSS regions (Supplementary Fig. S6) were fixed exclusively in broilers while they were either undetected or segregating in layer groups indicating their possible association with broiler-related traits. Almost one-third (*n* = 28) of these variants resided within the largest broiler pSS region (B-R09 in Supplementary Fig. S5) on chromosome1 and represented genes: *PARPBP, PMCH, NUP37, CCDC53, GNPTAB, SYCP3 and CHPT1*. This region also harboured the *IGF1* gene, which has known effect on growth, but the single pfVar from this gene (an intronic-MCE variant) was prevalent not only in broilers but also in layers in the present study. All these genes from B-R09 region were also detected within sweep regions in previous studies analysing commercial broiler lines.^26,41^

The rest of the broiler-specific SNPs were either intergenic or belonged to a few genes, namely, *ROBO2, AMMECR1, BMPR1B, SPRED1, MEIS2, ARFGEF2, USE-1 and MYO9B.* The *MYO9B* (*Myosin IXB*) gene is interesting as, in human, variants within this gene have been found associated with inflammatory bowel disease, ulcerative colitis and coeliac disease.^59,60^ In our study, the pSS region (B-R60 in Supplementary Fig. S5) harbouring this gene coincided with QTLs associated with antibody responses to *Escherichia coli* and *Salmonella enteritidis*, and growth*.* We detected one broiler-specific non-synonymous TOL SNP and several other ubiquitously present intronic-MCE variants from this gene. Based on the information from human studies, we speculate that this gene might have roles in triggering antibody responses to *E. coli* and *Salmonella* in chicken. Moreover, being a myosin encoding gene, the broiler-specific variant(s) within this may also affect muscle growth.

A number of layer-specific variants were also discovered—some of which were fixed exclusively in either BEL or WEL lines while the rest were fixed in both the layer groups. The variants fixed in both the layer groups (*n* = 20) came from nine genes only. Since direct effects of most of these genes are yet to be established, we investigated the overlapping QTLs to establish potential association of these genes with layer-related traits. For instance, a number of pfVars were detected from the gene *ADAMTS7* (*ADAM metallopeptidase with thrombospondin type 1 motif, 7*) including one non-synonymous INTOL mutation. We speculate that the variants within this gene may be associated with egg-shell strength in chicken as one member of the *ADAMTS* gene family (*ADAMTS1*) has previously been shown to be up-regulated in the uterus of hens laying hard-shelled eggs compared with those laying soft-shelled eggs.^61^ The region harbouring the *ADAMTS7* gene overlapped with QTLs for several traits including two for egg-shell strength and stiffness.^62^ Nevertheless, since the members of this gene family are known to perform a variety of physiological roles by encoding enzymes with protease activities, the *ADAMTS7* genes may have wider effects on multiple traits.^63^

The genes, *DOCK2* (*dedicator of cytokinesis 2*) and *GFPT2* (*glutamine-fructose-6-phosphate transaminase 2*), were detected from two pSS regions on Chromosome13 and coincided with QTL(s) associated with age-at-first egg and body weight.^64^ Both the genes contained several layer-specific and some ubiquitous variants. Since age-at-sexual- maturation is correlated to body weight, these genes possibly have pleiotropic effects on these traits, with the fixed pfVars probably conferring later maturity in layers.

A number of genes with BEL-specific variants on chromosome 6 coincided with a QTL suggestively associated with egg shell colours^65^ and these include: *FRMPD2* (containing 1 stop-gain, 2 non-syonymous TOL and 11 intronic-MCE-SNPs)*, HIF1AN* (1 intronic-MCE variant), *MYPN* (1 non-syonymous TOL SNP) and *ATOH7* (2 RNA structure-altering SNPs). The brown colour of egg shell derives from a major pigment called protoporphyrin, which is secreted from the surface epithelial cells of the uterus and is deposited onto the shell.^66^ The shell colouration can be affected by a number of mechanisms that control either the production or precipitation of the protoporphyrin.^67^ One recent study has suggested that the brown and white egg layers are not dissimilar in the protoporphyrin content, but it is the control mechanism affecting the secretion and deposition of the pigment that causes the difference in egg colour.^66^ Although no direct evidence was found in the extant literature linking any of the above genes with egg shell colour, but the *FRMPD2* (*FERM and PDZ domain containing 2*) appeared to be a strong candidate as a regulator of protoporphyrin precipitation. This is because it encodes a peripheral membrane protein that binds phosphatidylinositol 3,4-bisphosphate, which in turn plays important role in membrane trafficking.^68,69^ This gene, can therefore, play a regulatory role in the secretion and deposition of protoporphyrin on egg shell.

## 4. Concluding remarks

This paper reports the discovery and characterization of over 15 million SNPs from the chicken genome with the goal to delineate those with potential functional consequences—either having adaptive advantage or deleterious effect. To our knowledge, this is so far the largest study of its kind on chicken not only because a huge number of variants have been detected but also because a large number of populations and individuals that have been screened. Moreover, a number of complementary approaches were adopted to identify putatively functional variants from both coding and non-coding part of the genome. By investigating local fixation in the genome, this study has also detected a number of putative sweep regions and candidate casual variants therein. This analysis has confirmed a number of previously known sweep regions such as those involving the *BCDO2* gene for yellow skin colour and the *TSHR* gene and has identified novel regions, e.g. those potentially associated with bone strength and morphology, antibody response to *E. coli and Salmonella*, egg-shell colour and shell strength.

In spite of the discovery of millions of SNPs, the present study failed to capture the rare and low-frequency variants (AAF < 0.05)—a major limitation of adopting Pool-seq approach with low coverage. Low-frequency variants typically represent recent mutations and are often private to specific populations. Their detection requires screening of hundreds of individuals from each population, which was beyond the scope of the present study. Unlike in human, where discovery of rare disease-causing variants has medical significance, for farm animals it is unlikely to be a priority, unless a common disease is caused by many rare variants as proposed by ‘rare allele model’.^70^ Instead, for farm animals the priority is to improve commercially important traits through selective breeding and unless a disease is common enough it is unlikely to be a major focus in breeding programmes. Therefore, we argue that even though, low-frequency variants could not be characterized in the present study, it is the common variants that would have major application in breeding programmes of chicken.

Although the present study made efforts to characterize as many non-coding pfVars as possible using various approaches—viz. finding variants within conserved loci and known ncRNAs, predicting their effects on RNA structure and characterizing high-frequency variants within putative sweep regions—still the effort fell short due to incomplete annotation of chicken genome for functional non-coding elements. Knowledge of these elements, however, is rapidly improving due to advancements in transcriptome profiling (e.g. RNA-seq) and our predictive abilities of their functions through *in silico* analyses. Better characterization of these variants will therefore be possible in near future.

In conclusion, it is expected that by providing a large catalogue of variants in general and potentially functional variants in particular, this study will have major implications in breeding programmes and future research in chicken in the areas of genomic selection, genome-wide association studies, fine mapping of QTL, haplotype mapping and locating candidate causal variants.

## Supplementary data

Supplementary data are available at www.dnaresearch.oxfordjournals.org.

## Funding

This work was funded by a BBSRC/DEFRA LINK grant. C.B.'s contribution was sponsored by a fellowship from the National Council for Scientific and Technological Development – Brazil (CNPq, grant 201306/2010-7). Funding to pay the Open Access publication charges for this article was provided by the Research Council UK (RCUK).

## Supplementary Material

Supplementary Data
