# Supplementary material for: Functional classification of 15 million SNPs detected from diverse chicken populations
Source: DNA Res. 2015 Apr 29;22(3):205–17. doi: 10.1093/dnares/dsv005 (PMC4463845; doi:10.1093/dnares/dsv005)
Supplement: Supplementary Data [file supp_22_3_205__index.html]

Functional classification of 15 million SNPs detected from diverse chicken populations — Supplementary Data 

# Functional classification of 15 million SNPs detected from diverse chicken populations

## Supplementary Data

Supplementary Data

**Files in this Data Supplement:**

- Supplementary Data - Doc file
- Supplementary Figure 1 - tif file
- Supplementary Figure 2 - tif file
- Supplementary Figure 3 - tif file
- Supplementary Figure 4 - tif file
- Supplementary Figure 5 - png file
- Supplementary Figure 6 - png file
