# Supplementary material for: Functional classification of 15 million SNPs detected from diverse chicken populations
Source: DNA Res. 2015 Apr 29;22(3):205–17. doi: 10.1093/dnares/dsv005 (PMC4463845; doi:10.1093/dnares/dsv005)
Supplement: Supplementary Data [file supp_dsv005_dsv005supp.doc]

# Supplementary Information

## Materials and Methods

### SNP detection from NGS data

Variant detection using the “*mpileup*” function of the SAMtools (v0.1.18) package[^1^](#_ENREF_1) was performed using the following commands:

samtools mpileup **–**q20 **–**Q20 **-**AB **-**ugf <referenceFile.fa> <bamFile.bam> | bcftools view **-**bvcg > <var.raw.bcf>

bcftools view <var.raw.bcf> | vcfutils.pl varFilter **-**D99999 > <var.flt.vcf>

In the above commands –q and –Q options were used to set the thresholds for mapping and base qualities to be at least 20. The option –A allowed using all the reads including anomalous read pairs. The option B was used to disable computation of Base Alignment Quality (BAQ), which, by default, is turned on when the *mpileup* command is used. While BAQ calculation can help reduce the calling of FPs due to incorrect alignments near small indels, there have been recent suggestions that BAQ may be too strict causing real SNPs to be missed[^2^](#_ENREF_2) Moreover, BAQ computation can be computationally very demanding for large dataset as ours. The –D option with bcftools allowed setting a maximum threshold on the depth of sequence coverage that would be analysed.

### FDR estimation

For estimation of FDR, initially 28 regions were randomly selected from chicken genome. Primers were designed and the regions were sequenced on the same 10 individuals from RI-J line using Sanger method that were previously sequenced by NGS. The randomly chosen regions represented Chromosomes *GGA:* 1-6, 10-12, 15-19, 21, 22, 24, 26 and 28 and contig, LGE22C19W28_E50C23. Sanger sequences were mapped to chicken reference genome using BWA long read mapping algorithm (*bwasw*) using default options and SNPs were called using SAMtools *mpileup* function. After manual inspection only regions that produced good quality sequence from all 10 individuals were analysed. Three regions were excluded from FDR analyses, because in two of those, good quality Sanger data from all 10 individuals could not be generated even after repeated attempts, while in the third one the sequences mapped to the genome with poor quality score. Some of the NGS-SNPs detected from this third region had a very high coverage indicating that this region might contain duplicated sequence.

## References

## Supplementary Tables

### Table S1: Estimation of false discovery rates based on two filtration methods

| SNPQ | No. of TPs* | No. of FNs* | No. of FPs* | No.  of TNs * | Sensitivity [TP/(TP + FN)] | Specificity [(TN/(TN + FP)] | FPR (1- specificity) | FNR (1 - sensitivity) | Error rate in NGS (proportion of FPs) |
| --- | --- | --- | --- | --- | --- | --- | --- | --- | --- |
| ***Method1: Filtration based on SNPQ*** | | | | | | | | | |
| Q≥0 | 97 | 0 | 103 | 9988 | 1 | 0.99 | 0.01 | 0.00 | 0.52 |
| Q≥10 | 97 | 0 | 75 | 10016 | 1 | 0.99 | 0.01 | 0.00 | 0.44 |
| Q≥20 | 97 | 0 | 46 | 10045 | 1 | 1.00 | 0.00 | 0.00 | 0.32 |
| Q≥30 | 97 | 0 | 25 | 10066 | 1 | 1.00 | 0.00 | 0.00 | 0.20 |
| Q≥40 | 97 | 0 | 14 | 10077 | 1 | 1.00 | 0.00 | 0.00 | 0.13 |
| Q≥50 | 96 | 1 | 8 | 10083 | 0.99 | 1.00 | 0.00 | 0.01 | 0.08 |
| Q≥60 | 91 | 6 | 4 | 10087 | 0.94 | 1.00 | 0.00 | 0.06 | 0.04 |
| ***Method2: Filtration based on SNPQ +evidence of alternative allele by at least 1 forward strand and 1 reverse strand reads*** | | | | | | | | | |
| Q≥0 | 95 | 2 | 34 | 10057 | 0.98 | 1.00 | 0.00 | 0.02 | 0.26 |
| Q≥10 | 95 | 2 | 27 | 10064 | 0.98 | 1.00 | 0.00 | 0.02 | 0.22 |
| Q≥20 | 95 | 2 | 12 | 10079 | 0.98 | 1.00 | 0.00 | 0.02 | 0.11 |
| Q≥30 | 95 | 2 | 6 | 10085 | 0.98 | 1.00 | 0.00 | 0.02 | 0.06 |
| Q≥40 | 95 | 2 | 3 | 10088 | 0.98 | 1.00 | 0.00 | 0.02 | 0.03 |
| Q≥50 | 94 | 3 | 2 | 10089 | 0.97 | 1.00 | 0.00 | 0.03 | 0.02 |
| Q≥60 | 90 | 7 | 1 | 10090 | 0.93 | 1.00 | 0.00 | 0.07 | 0.01 |

* TP=True Positive and refers to SNPs detected by NGS and also confirmed by Sanger ; FN= False Negative and refers to SNPs detected by Sanger method only; FP=False Positive and refers to SNPs that were detected by NGS method only and were confirmed by Sanger; TN= True Negatives and refers to sequenced bases that were not called as SNPs by neither of the methods

### Table S2: Significant canonical pathways enriched for genes with high density of AA-altering variants. Genes with >10 AA-altering SNPs per kb of coding sequence were analysed.

| **Significant canonical pathways** | **B-H corrected *P* -value** | **Ratio*** | **Top functions and associated diseases** |
| --- | --- | --- | --- |
| cAMP-mediated signalling | 9.62E-03 | 16/226 (0.071) | DNA Replication, Recombination, and Repair; Cellular Assembly and Organization; Cellular Function and Maintenance |
| Basal cell carcinoma signalling | 2.93E-02 | 8/78 (0.103) | Cancer; Dermatological Diseases and Conditions; Embryonic Development |
| Chondroitin sulphate  Biosynthesis (late stages) | 4.74E-02 | 6/55 (0.109) | Carbohydrate Metabolism; Small Molecule Biochemistry; Cell-To-Cell Signaling and Interaction |
| G-protein coupled receptor Signalling | 4.77E-02 | 15/276 (0.054) | DNA Replication, Recombination, and Repair; Cellular Assembly and Organization; Cellular Function and Maintenance |
| Glycine betaine degradation | 4.99E-02 | 3/23 (0.13) | Amino Acid Metabolism; Molecular Transport; Small Molecule Biochemistry |
| Chondroitin sulphate biosynthesis | 4.99E-02 | 6/72 (0.083) | Carbohydrate Metabolism; Small Molecule Biochemistry; Cell-To-Cell Signaling and Interaction |
| L-serine degradation | 4.99E-02 | 2/6 (0.333) | Amino Acid Metabolism; Neurological Disease; Small Molecule Biochemistry |
| Gai signalling | 4.99E-02 | 9/135 (0.067) | DNA Replication, Recombination, and Repair; Cancer; Cellular Assembly and Organization |
| Dermatan sulphate biosynthesis | 4.99E-02 | 6/73 (0.082) | Carbohydrate Metabolism; Small Molecule Biochemistry; Cellular Assembly and Organization |

*The ratio is calculated as: (Number of genes in a given pathway that meet cut-off criteria)/ total number of genes that make up that pathway)

### Table S3: Significant canonical pathways enriched for genes with non-synonymous deleterious SNPs as predicted by SIFT or PROVEAN packages

| Groups | Number of pathways significantly enriched with DEL SNPs* | Number (%) of signalling pathways | Top five significant pathways |
| --- | --- | --- | --- |
| Broiler | 191 | 167 (87%) | 1. Axonal Guidance Signalling 2. Molecular Mechanisms of Cancer 3. Glioblastoma Multiforme Signalling 4. PTEN Signalling 5. Thrombin Signalling |
| Brown Egg Layer (BEL) | 162 | 149 (92%) | 1. Axonal Guidance Signalling 2. Molecular Mechanisms of Cancer 3. Glioblastoma Multiforme Signalling 4. Neuropathic Pains Signalling in Dorsal Horn Neurons 5. Glioma Signalling |
| White Egg layer (WEL) | 211 | 193 (91%) | 1. Axonal Guidance Signalling 2. Glioblastoma Multiforme Signalling 3. Thrombin Signalling 4. PTEN Signalling 5. Signalling by Rho Family GTPase |
| Inbred | 2 | 2 (100%) | 1. Role of BRCA1 in DNA Damage Response 2. DNA Double-Strand Break Repair by Non-Homologous End Joining |

*Significant after correction for B-H multiple testing

### Table S4: Result from RNAsnp analysis showing the number (and %) of variants from different categories that were predicted to affect RNA secondary structure

| **Analysis mode** | **UTR** | **Non-synonymous** | **Synonymous** | **ncRNA** |
| --- | --- | --- | --- | --- |
| ***At P-value ≤ 0.05*** | | | | |
| Mode1 d_max | 9,304 (4.52%) | 8,791  (4.80%) | 5,368  (3.51%) | 112 (3.57%) |
| Mode1 r_min | 4,462 (2.16%) | 4,520  (2.46%) | 3,017  (1.97%) | 30  (0.96%) |
| Mode1 d_max and r_min | 4,955 (2.41%) | 4,736  (2.59%) | 2,918  (1.90%) | 55  (1.76%) |
| Mode2 | 9,470 (4.61%) | 10,128 (5.53%) | 5,860  (3.83%) | 146 (4.66%) |
| Mode1 or 2 | 19,389 (9.43%) | 19,686 (10.75%) | 12,056 (7.89%) | 219 (6.99%) |
| ***At P-value ≤ 0.01*** | | | | |
| Mode1 d_max | 670  (0.33%) | 449  (0.25%) | 315  (0.21%) | 23  (0.73%) |
| Mode1 r_min | 483  (0.23%) | 363  (0.20%) | 220  (0.14%) | 17  (0.54%) |
| Mode1 d_max and r_min | 95  (0.05%) | 61  (0.03%) | 46  (0.03%) | 10  (0.32%) |
| Mode2 | 1,836 (0.89%) | 1,862  (1.02%) | 1,015  (0.66%) | 94  (3.00%) |
| Mode 1 or 2 | 2,666 (1.30%) | 2,570  (1.40%) | 1,406  (1.74%) | 87  (2.78%) |

### Table S5: Summary of selection signature analyses in different chicken groups

|  | **Broiler** | **BEL** | **WEL** |
| --- | --- | --- | --- |
| No. of analysable windows | 45,174-45,378 | 44,237-45,180 | 44,577-45,357 |
| Average SNP count in analysed windows (± SD) | 202 ± 84 | 201 ± 85 | 185 ± 83 |
| Average Hp in analysed windows | 0.35 ± 0.09 | 0.30 ± 0.12 | 0.26 ± 0.13 |
| Range of average Hp in chromosomes | 0.29-0.43 | 0.20-0.40 | 0.16-0.39 |
| Variation in chromosome-wise critical Hps at P<0.05 | 0.056-0.341 | 0.020-0.312 | 0.011-0.231 |
| Variation in chromosome-wise critical Hps at P<0.01 | 0.028-0.329 | 0.010-0.296 | 0.003-0.200 |
| Variation in chromosome-wise critical Hps at P<0.001 | 0.015-0.306 | 0.004-0.274 | 0.002-0.074 |
| Number of significant windows (P<0.05) common across all lines within a group | 143 | 163 | 49 |
| Number of non-overlapping regions with signals of selective sweep (after merging the overlapping or adjacent windows) | 60 | 66 | 25 |
| Size of largest region of putative sweep and chromosome | 260 Kb in GGA1 | 260 Kb in GGA8 | 200 kb in GGA13 |

## Supplementary Figure Legends

**Figure S1: Distributions of SIFT (a) and PROVEAN (b) scores for nonsynonymous SNPs.** Arrows show the default threshold values below which the variants were predicted to be intolerant. The SIFT scores were collated into 40 equal intervals spanning the range from 0 to 1. PROVEAN scores were collated into 30 equal intervals spanning the ranges from -15 to 15.

**Figure S2. Bar diagram showing the proportions of evolutionary intolerant nonsynonymous SNPs detected from one or more sequenced lines.** The Intolerant variants were predicted using the SIFT or PROVEAN packages.

**Figure S3(a-d). Frequency distribution of non-reference or alternative alleles of SNPs within most conserved elements and non-conserved regions of genome in different chicken groups.** X axis represents the allele frequency (ranges from 0 to 1) and Y axis represents percentage of SNPs within a category.

**Figure S4. Relative positions of stopgain SNPs within proteins**. Relative position was calculated by dividing the SNP position in protein with the length of the protein. Position near 0 indicates that the SNP is located near N-terminus and position near 1 indicates location near C-terminus.

**Figure S5. Heat map showing the putative selective sweep windows/regions detected from at least one of the three chicken groups.** B=broiler, BEL=brown egg layer and WEL=White egg layer. The colour gradient from yellow to green indicate scores from 0 to 3 based on the level of significance, where the score 0 stands for “not significant at *P*<0.05” whereas score 3 stands for “highly significant at *P*<0.001”. The columns at the right hand side of the heat map represent the genomic location of the 40kb windows and the region-IDs assigned to these windows. Adjacent or overlapping windows within a chicken group have same region IDs. The heat map was generated by the Genesis program (<http://genome.tugraz.at/>).

**Figure S6. Heat map based on the allele frequency of putative functional variants fixed within at least one of the three groups of chicken.** B=broiler, BEL=brown egg layer and WEL=White egg layer. The colour gradient from yellow to green indicates non-reference or alternative allele frequency from 0 to 1. The data columns at the right side of the heat map are: SNPID (chr:pos), sweep region IDs, SNP annotation, and associated gene(s). The heat map was generated by the Genesis program (<http://genome.tugraz.at/>).
