# Supplementary figures and images for: Functional classification of 15 million SNPs detected from diverse chicken populations
Source: DNA Res. 2015 Apr 29;22(3):205–17. doi: 10.1093/dnares/dsv005 (PMC4463845; doi:10.1093/dnares/dsv005)

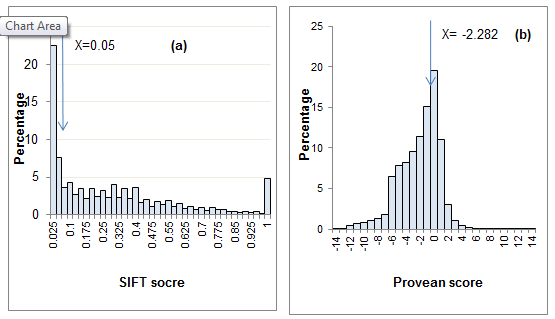

Supplement: Supplementary Data [file supp_dsv005_dsv005supp_fig1.tif]

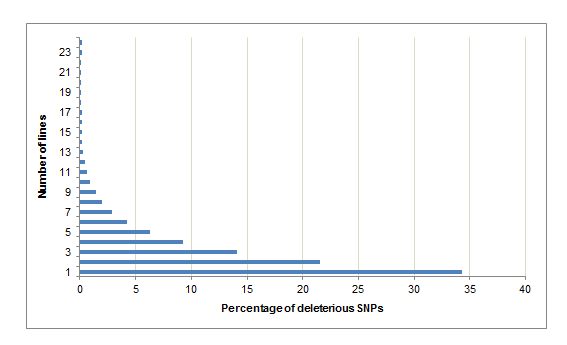

Supplement: Supplementary Data [file supp_dsv005_dsv005supp_fig2.tif]

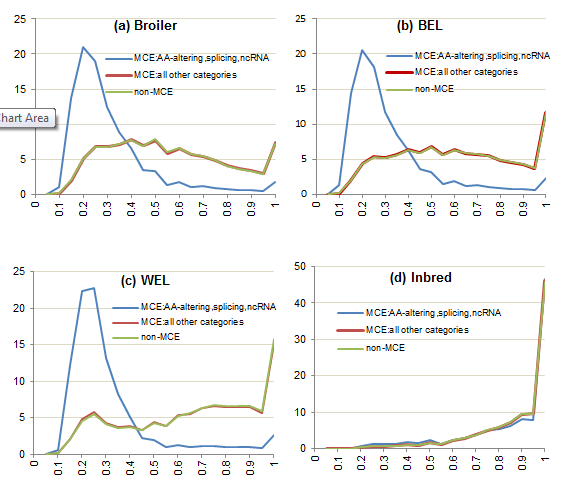

Supplement: Supplementary Data [file supp_dsv005_dsv005supp_fig3.tif]

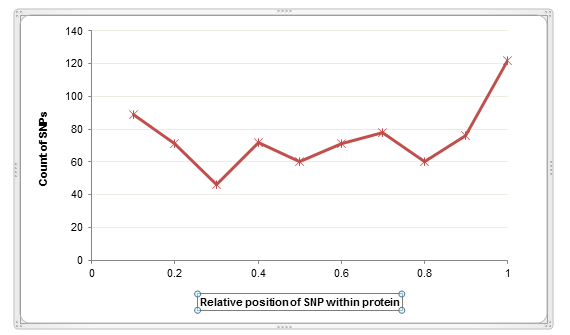

Supplement: Supplementary Data [file supp_dsv005_dsv005supp_fig4.tif]

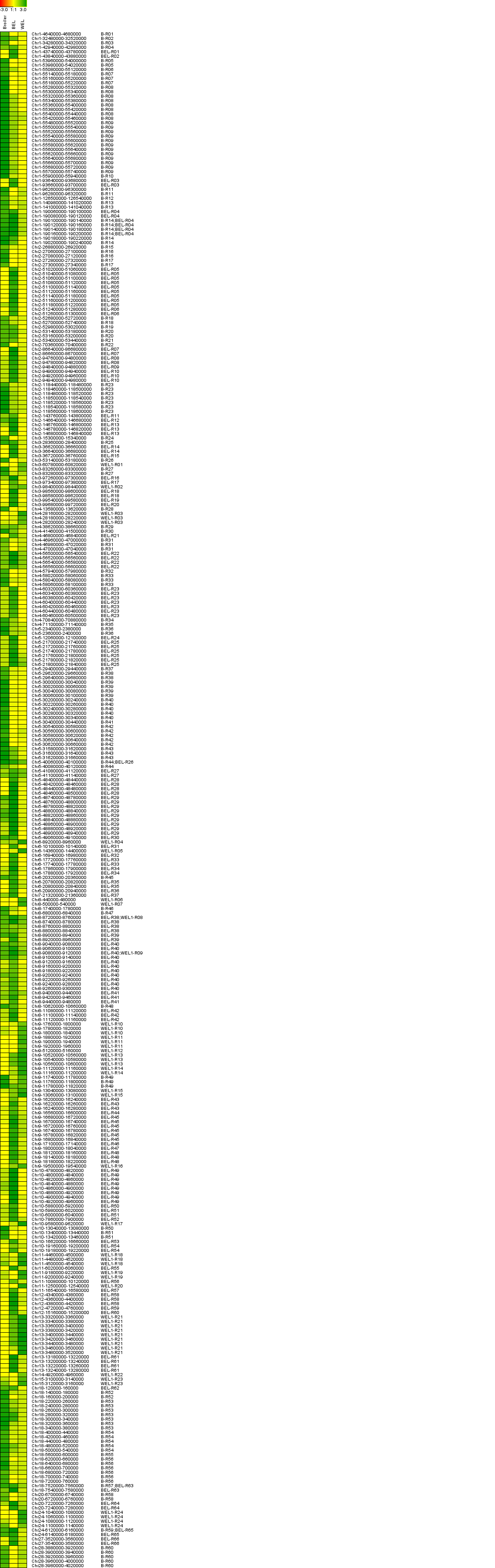

Supplement: Supplementary Data [file supp_dsv005_dsv005supp_fig5.png]

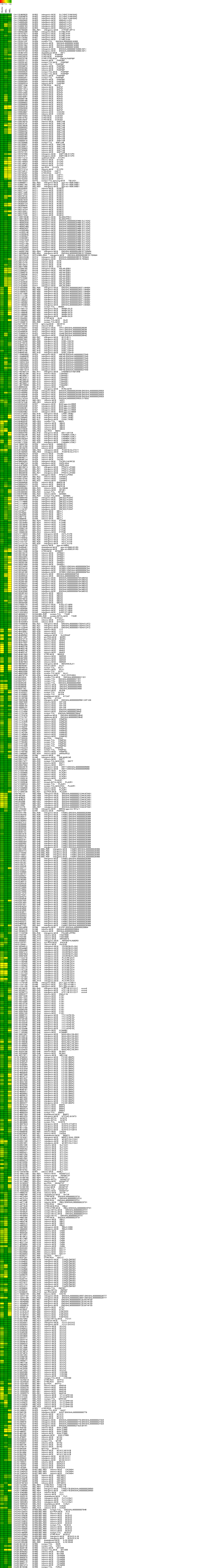

Supplement: Supplementary Data [file supp_dsv005_dsv005supp_fig6.png]
